# Chinese Herbal Medicine as an Adjunctive Therapy for Breast Cancer: A Systematic Review and Meta-Analysis

**DOI:** 10.1155/2016/9469276

**Published:** 2016-05-09

**Authors:** Libing Zhu, Lingru Li, Yingshuai Li, Ji Wang, Qi Wang

**Affiliations:** Center of Reproductive Medicine and Traditional Chinese Medicine Constitution, School of Preclinical Medicine, Beijing University of Chinese Medicine, Beijing 100029, China

## Abstract

Chinese herbal medicine (CHM) has been widely used as an adjunctive therapy for breast cancer, while its efficacy remains unexplored. The purpose of this study is to evaluate the efficacy of CHM combined with chemotherapy for breast cancer. The study results showed that CHM combined with chemotherapy significantly increased tumor response and KPS as compared to using chemotherapy alone (RR = 1.36; 95% CI = 1.24–1.48; *P* < 0.00001; RR = 1.38; 95% CI = 1.26–1.52; *P* < 0.00001, resp.). Besides, CHM as an adjunctive therapy significantly reduced the nausea and vomiting at toxicity grade of III–IV (RR = 0.37; 95% CI = 0.27–0.52; *P* < 0.00001). Moreover, the combined therapy significantly prevented the decline of WBC in patients under chemotherapy at toxicity grade of III–IV (RR = 0.49; 95% CI = 0.34–0.69; *P* < 0.00001) and prevented the decline of platelet at toxicity grade of III–IV or I–IV (RR = 0.29; 95% CI = 0.12–0.73; *P* = 0.008; RR = 0.77; 95% CI = 0.63–0.94; *P* = 0.009, resp.). This study suggests that CHM combined with chemotherapy in comparison with chemotherapy alone can significantly enhance tumor response, improve KPS, and alleviate toxicity induced by chemotherapy in breast cancer patients. However, a firm conclusion could not be reached due to the lack of high quality trials and large-scale RCTs, so further trials with higher quality and larger scale are needed.

## 1. Introduction

Breast cancer is the most common malignancy and the leading cause of cancer mortality in women worldwide [1]. American Cancer Society (ACS) estimates that there will be 246,660 cases of women diagnosed with breast cancer in US and 40,450 women die of the disease during 2016 [2]. The prognosis of newly diagnosed breast cancer patients is determined by the classification of breast cancer. There are at least four main subtypes of breast cancer according to different patterns of gene expression [3, 4]. Luminal A tends to have the best prognosis, which comprises estrogen receptor-positive (ER+) and/or progesterone receptor-positive (PR+), human epidermal growth factor receptor 2-negative (HER2−), and grade 1 or 2 tumors [3]. Luminal B includes ER+ and/or PR+, HER2+ or HER2−, and grade 3 tumors. The other 2 subtypes confer bad prognosis, which contain the HER2 overexpressing breast cancer (ER−, PR−, and HER2+) and the triple-negative breast cancer (ER−, PR−, and HER−) [4]. Nowadays the standard treatment options for patients with breast cancer include surgery, chemotherapy, radiotherapy, and endocrine therapy [5]. Usually, endocrine therapy remains the core adjuvant therapy for most of the early breast cancer patients who are diagnosed as ER+, while chemotherapy is recommended as the first-line systemic adjuvant modality for most HER2+ and triple-negative breast cancer patients [6]. Unfortunately, even curing surgery may accompany the risk of recurrence and metastasis, patients may produce resistance to chemotherapy, and these treatments can cause serious side effects in short or long term [6, 7].

Complementary and alternative medicine (CAM) is widely used by 50% cancer patients, and patients with breast cancer use CAM more frequently than others which is accounting for 63 to 83% [8–12]. The types of CAM used by patients with breast cancer vary between countries. In US and Europe, naturopathy and homeopathy are the most commonly used CAM types, whereas for patients with Chinese background, Chinese herbal medicine (CHM) tends to be the most popular type among cancer patients and 86.4% breast cancer patients used CHM for treatment [5, 9, 13, 14]. CHM as an adjuvant therapy to conventional therapy (mainly refers to chemotherapy) has been commonly used to prolong survival time of cancer patients, alleviate clinical symptoms, and minimize the adverse events caused by chemotherapy in Asia [15]. Previous clinical studies suggested that CHM adjuvant therapy might have potential roles in three main parts. First of all, it can improve the quality of life (QoL) and prolong the survival time [16]. Next, it may improve the immune function of breast cancer patients and prevent metastasis and recurrence [17, 18]. Finally, it can prevent or reduce toxicity from anticancer agents and enhance the effect of anticancer agents [16, 17]. However, there is lack of systematic review to assess the potential efficacy of CHM as an adjuvant therapy for breast cancer. Thus, the aim of this study is to conduct a systematic review to evaluate the efficacy of CHM combined with chemotherapy for breast cancer, using evidence from randomized controlled trials (RCTs) upon tumor response, immune function, adverse events, and QoL.

## 2. Methods

RCTs were retrieved from nine databases, theses, and conference papers by using electronic method as well as manual method. LBZ and LRL reviewed these studies independently. The first reviewer extracted the data from included studies and the second reviewer verified them again. Two reviewers rectified the discrepancies according to the original articles. If the consensus still cannot be achieved, a third party (QW) were sought for advising. Studies can be included in the meta-analysis only when they satisfied the criteria.

### 2.1. Search Strategy

The following databases were retrieved without any language restriction: PubMed, Cochrane Central Register of Controlled Trials, ISI Web of Science, Scopus, CINAHL Plus (EBSCO), EMBASE, China Journal Full-Text Database, China National Knowledge Infrastructure (CNKI), and Chinese Scientific Journal Database. Publications available from the inception of databases to January 2016 were reviewed to find out the appropriate RCTs of CHM for breast cancer. The following terms were searched in the databases: (Traditional Chinese Medicine OR Chinese Medicine OR traditional herbal medicine OR Chinese herbal medicine OR Chinese herbal drug OR herbal medicine OR traditional Japanese medicine OR traditional medicine OR materia medica OR Oriental medicine OR herb OR medicinal plant OR medicinal herbs OR medicinal plant product OR plant extract OR plant preparation OR herbal preparation OR phytotherapy OR herb therapy OR alternative medicine OR alternative therapy OR complementary therapy OR complementary medicine OR complementary and alternative therapy) AND (breast cancer OR breast carcinoma OR mammary cancer OR breast tumor) AND (clinical trial OR phase 1 clinical trial OR phase 2 clinical trial OR phase 3 clinical trial OR phase 4 clinical trial OR controlled clinical trial OR randomized controlled trial). The above terms in Chinese were searched in Chinese databases.

### 2.2. Inclusion Criteria

Only studies which meet all of the following criteria can be included in the meta-analysis. (1) Participants: participants are postoperative breast cancer patients and patients treated by chemotherapy. (2) Type of study: only RCTs were eligible. (3) Type of intervention: studies compared chemotherapy combined with or without CHM. For studies using other agents as the third arm, only the two arms using CHM with or without chemotherapy can be included for meta-analysis. (4) Type of outcome measurement: tumor response and Karnofsky performance score (KPS) were the main outcome measurements; other outcome measurements which contained immunoregulation and reduction in adverse events of chemotherapy were also considered.

### 2.3. Exclusion Criteria

Studies were excluded if they did not meet the above inclusion criteria. Additionally, trials with one or more of the following conditions were also excluded: (1) nonoriginal research such as review articles or letter to the editors; (2) duplicated publications of other studies; (3) CHM which were used in both treatment group and control group.

### 2.4. Outcome Measures

Tumor response of CHM on the number of breast cancer patients with complete response (CR) or partial response (PR), as well as those with progressive disease (PD) according to the WHO scale, was investigated [19]. The disappearance of all known tumor lesions is considered as CR, 50% or more reduce in total tumor size of the lesions is considered as PR (determined by two observations not shorter than 4 weeks apart), and PD refers to 25% or more increase in total tumor size of the lesions or the appearance of new lesions. The improved or stable performance status of subjects based on KPS was also examined, in which score of 100 refers to a normal subject without any complaints, score of 70 refers to a patient who is unable to carry on normal activity, score of 50 refers to a patient requires considerable assistance, score of 40 refers to a disabled patient, and score of 30 refers to a patient who is hospitalization-recommended [20]. The efficacy of CHM on relieving the adverse events caused by chemotherapy containing nausea and vomiting and reduction of white blood cell as well as platelet were studied by grading the acute and subacute toxic effects of cancer therapy [21]. The efficacy of CHM on immunoregulation includes the change of mean values of CD3 T cell level, CD4 T cell level, CD8 T cell level, and CD4/CD8 ratio.

### 2.5. Quality Assessment

Methodological quality of included studies was assessed by using the risk of bias tools in accordance with Cochrane Handbook version 5.1.0 [22]. Risk of bias for assessing the methodological quality of RCTs mainly included six items: selection bias (random sequence generation and allocation concealment), performance bias and detection bias (blinding), attrition bias (incomplete outcome data), reporting bias (selective reporting), and other bias. Each item was ranked as low, high, and unclear risk. The methodological quality of all trials was assessed as the following three levels: low risk of bias (all items were ranked as low risk), unclear risk of bias (at least one item was ranked as unclear risk), or high risk of bias (at least one item was ranked as high risk). At least two reviewers assessed the all trials and any disagreements were solved by the third reviewer consensus.

### 2.6. Data Analysis

Cochrane Collaboration Review Manage software (RevMan 5.2) was used for data analysis. Dichotomous data were reported as relative risk (RR) with 95% confidence intervals (95% CI) whereas continuous data were expressed as mean ± standard deviation (SD). *I* ^2^ was used to assess the heterogeneity; if the heterogeneity exists in the pooled studies (*I* ^2^ > 50%), a random-effect model was applied; otherwise the fix-effect model was applied [23]. The differences between the treatment groups and control groups were considered to be statistically significant when *P* < 0.05.

## 3. Results

### 3.1. Characteristics of the Included Studies

**Figure 1:**
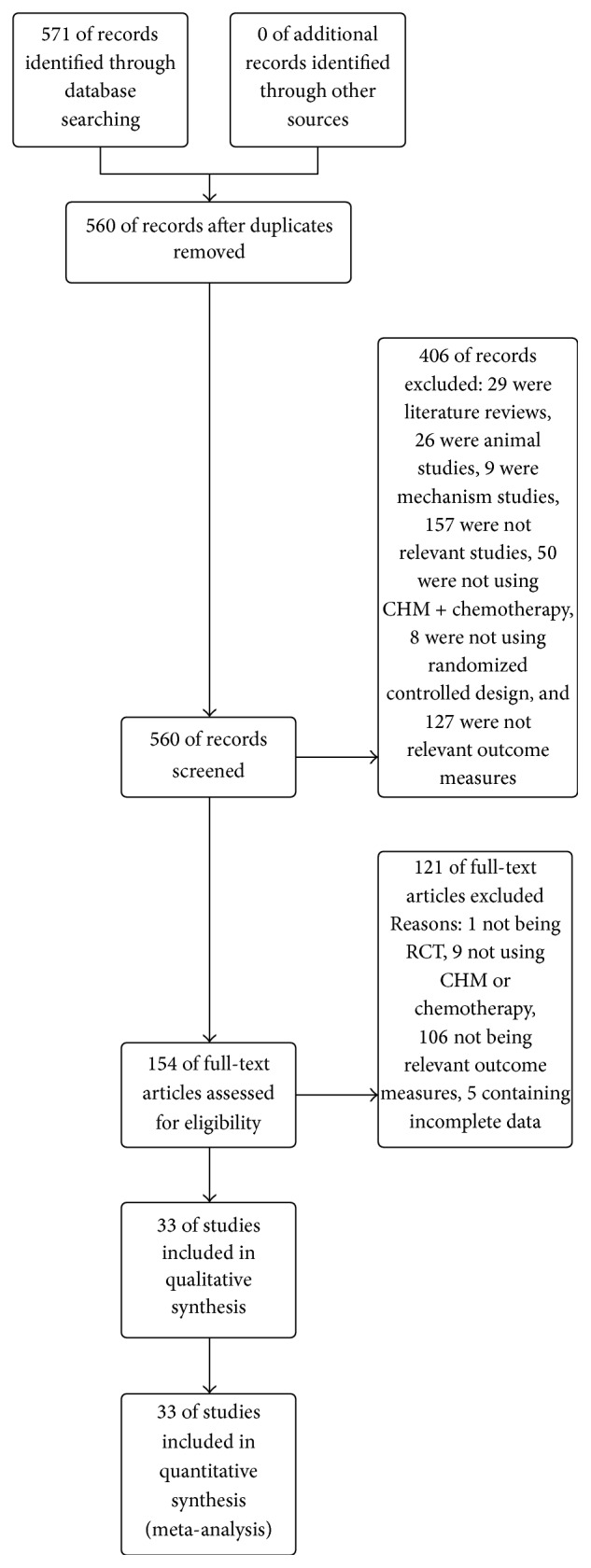
Study flow diagram.

**Figure 2:**
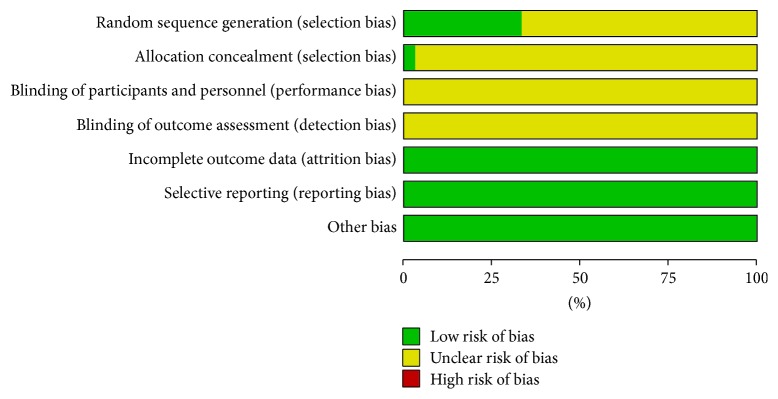
Risk of bias graph.

**Figure 3:**
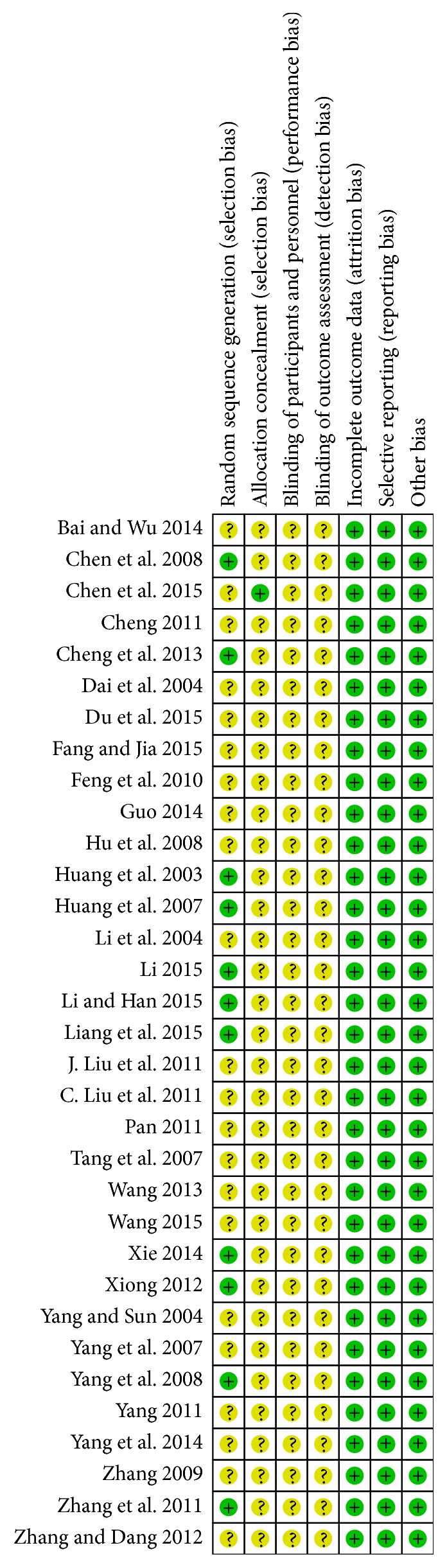
Risk of bias summary.

Overall, 571 studies were retrieved. There were 11 duplicated studies, 127 studies did not investigate outcome of interest (such as only investigated Chinese medicine syndrome scales), 29 studies were literature reviews, 26 studies were animal studies, 9 studies were mechanism studies, 157 were not relevant studies (such as investigated CHM for lymphedema after mastectomy), 50 studies were not using CHM combined with chemotherapy as intervention, and 8 studies were not RCTs. So only 154 studies satisfied the selection criteria, among which 106 studies did not investigate outcome of interest, 9 studies did not use CHM combined with chemotherapy as interventions, 1 study was not RCT, and 5 studies were with incomplete data. There were 33 RCTs included in this meta-analysis [16–18, 24–54] (Figure 1). A sum of 2098 patients was enrolled in these studies, at which 1066 patients participated in CHM combined chemotherapy and 1032 in chemotherapy (two patients dropped out from treatment group, one patient dropped out from control group, and other four patients withdraw or dropped out but did not report the specific number in each group). All patients recruited in the 33 studies were postoperative breast cancer patients, and basically all of the included studies can be evaluated as low risk of bias [16–18, 24–54]. The risk of bias of all included studies was shown in Figures 2 and 3. The course of therapy varied from 2 to 24 weeks in included studies. A list of therapeutic approaches and outcome measurements in each study were listed in Table 1. All studies claimed that the baseline data were comparable containing age, TNM (tumor node metastasis) stage, or histopathology.

### 3.2. Tumor Response

**Figure 4:**
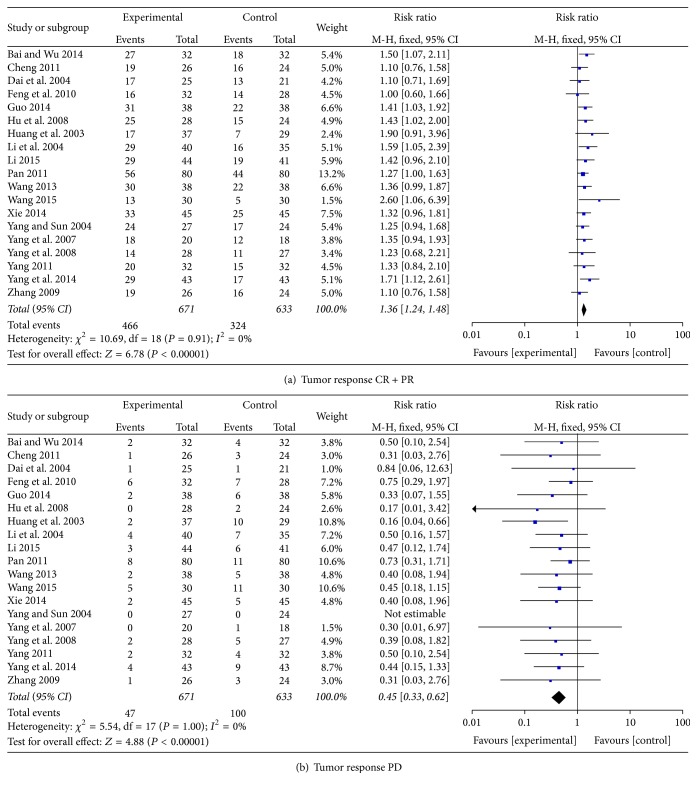
Tumor response.

Results from 19 studies showed that 69% (466/671) of patients using chemotherapy with CHM were reported as complete or partial response, while 51% (324/633) of patients only using chemotherapy were reported as complete or partial response, indicating that the treatment for breast cancer was significantly in favour of CHM combined with chemotherapy (RR = 1.36; 95% CI = 1.24–1.48; *P* < 0.00001) [24, 26, 28–31, 33, 36–38, 40–43, 46–50] (Figure 4(a)). 7% (47/671) of patients in the chemotherapy combined with CHM group were reported with progressive disease, while 16% (100/633) of patients without CHM were reported with progressive disease. Results from 19 studies showed that the combined treatment for breast cancer has a positive effect in the number of patients who reported progressive disease (RR = 0.45; 95% CI = 0.33–0.62; *P* < 0.00001) [24, 26, 28–31, 33, 36–38, 40–43, 46–50] (Figure 4(b)).

### 3.3. Performance Status

**Figure 5:**
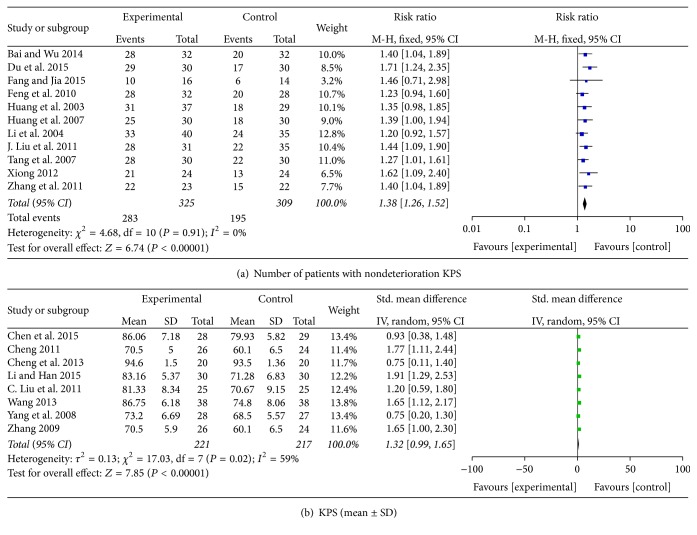
Quality of life: KPS.

The QoL changes on KPS were reported as two types of data in the included studies, the number of patients who reported the improved or stable performance status based on KPS (ten-point cutoff) and the mean ± SD of KPS before and after treatment. For the nondeterioration KPS, 11 studies of the 33 studies with evaluation of 634 patients were analyzed. There were 87% (283/325) of patients who reported nondeterioration in the combined therapy groups and in the chemotherapy groups it was 63% (195/309) [18, 27, 28, 34, 39, 40, 44, 45, 48, 50, 53]. Results from 11 studies showed that the combined using of chemotherapy and CHM was significantly related to improving QoL (RR = 1.38; 95% CI = 1.26–1.52; *P* < 0.00001) (Figure 5(a)). There was no significant heterogeneity among these studies (*I* ^2^ = 0%). The value of KPS was reported with pretreatment in seven studies [24, 25, 32, 38, 41, 42, 54] and posttreatment in eight studies [24, 25, 32, 33, 38, 41, 42, 54], and the pooled studies of pretreatment showed that there was no significant difference between combined therapy and chemotherapy alone (SMD = 0.18; 95% CI = −0.02–0.39; *P* = 0.07; *I* ^2^ = 0%). However, the pooled studies of posttreatment indicated significant difference between CHM combined with chemotherapy and chemotherapy alone (SMD = 1.32; 95% CI = 0.99–1.65; *P* < 0.00001; *I* ^2^ = 59%) (Figure 5(b)). Although *I* ^2^ is equal to 59%, dropping one of any of the studies did not change the result in favour of combined therapy.

### 3.4. Reduction in Chemotherapeutic Toxicity

**Figure 6:**
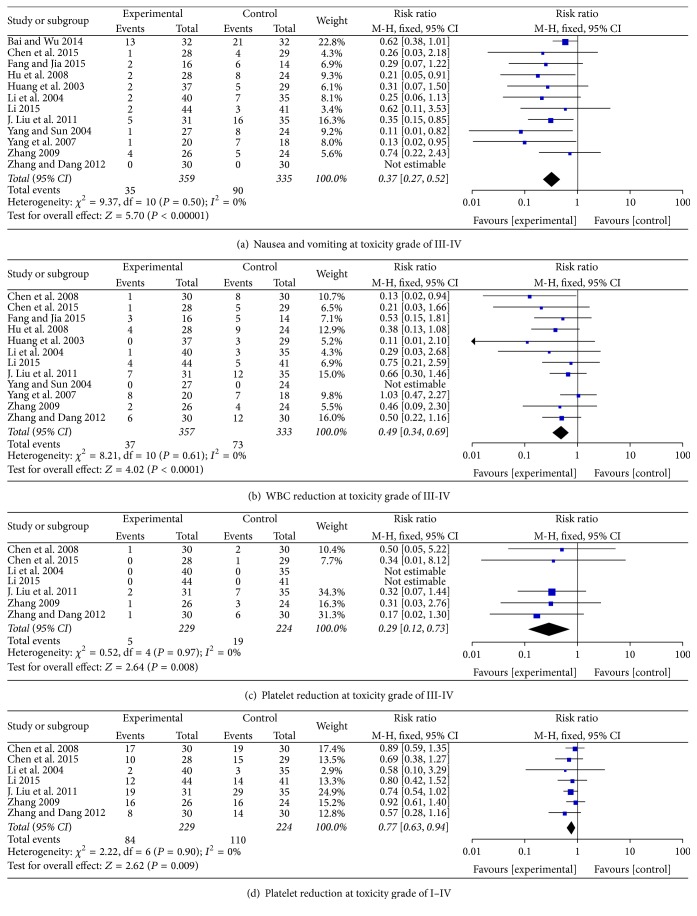
Reduction of adverse effects.

Nausea and vomiting are common adverse events of chemotherapy. There was significant reduction of nausea and vomiting at toxicity grade of III-IV in patients treated by CHM combined with chemotherapy (RR = 0.37; 95% CI = 0.27–0.52; *P* < 0.00001; twelve studies; 694 patients) [25, 27, 28, 35, 41, 43, 46, 48–50, 52, 53] (Figure 6(a)). However, the reduction of nausea and vomiting at toxicity grade of I–IV has significant heterogeneity (RR = 0.75; 95% CI = 0.69–0.82; *P* < 0.00001; *I* ^2^ = 91%) (data not shown). A significant reduction of WBC inhibition at toxicity grade of III-IV was found (RR = 0.49; 95% CI = 0.34–0.69; *P* < 0.00001; twelve studies; 690 patients) [16, 25, 27, 35, 41, 43, 46, 48–50, 52, 54] (Figure 6(b)). But there was significant heterogeneity in the studies with reduction of WBC inhibition at toxicity grade of I–IV (RR = 0.75; 95% CI = 0.69–0.82; *P* < 0.00001; *I* ^2^ = 91%) (data not shown). The decrease of platelet at the toxicity grade of III-IV or I–IV in patients with combined therapy was significantly reduced (RR = 0.29; 95% CI = 0.12–0.73; *P* = 0.008; seven studies; 453 patients; RR = 0.77; 95% CI = 0.63–0.94; *P* = 0.009; seven studies; 453 patients, resp.) (Figures 6(c) and 6(d)) [16, 25, 35, 41, 48, 52, 53].

### 3.5. Immunoregulation

**Figure 7:**
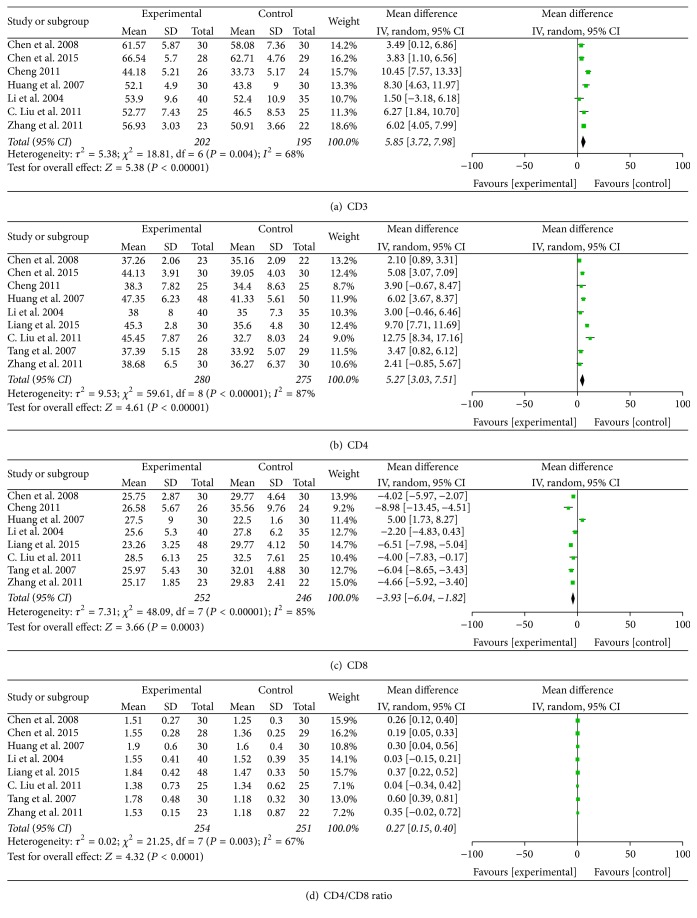
Immunoregulation.

There was a significant rise in CD3 T cell level in patients treated by chemotherapy combined with CHM. However, a significant difference of the heterogeneity test was found among the pooled seven studies (MD = 5.85; 95% CI = 3.72–7.98; *P* < 0.00001; *I* ^2^ = 68%) (Figure 7(a)) [16, 25, 38, 39, 45, 48, 54]. In addition, significant improvement in other immune effects also occurred in combined therapy group, including CD4 T cell level (MD = 5.27; 95% CI = 3.03–7.51; *P* < 0.00001; nine studies; 555 patients) (Figure 7(b)) [16, 17, 25, 38, 39, 44, 45, 48, 54], CD8 T cell level (MD = −3.93; 95% CI = −6.04–−1.82; *P* = 0.0003; eight studies; 498 patients) (Figure 7(c)) [14, 17, 38, 39, 44, 45, 48, 54], and CD4/CD8 ratio (MD = 0.27; 95% CI = 0.15–0.40; *P* < 0.0001; eight studies; 505 patients) (Figure 7(d)) [16, 17, 25, 39, 44, 45, 48, 54]. However, the heterogeneity test of CD4 T cell level (*I* ^2^ = 87%), CD8 T cell level (*I* ^2^ = 85%), and CD4/CD8 ratio (*I* ^2^ = 67%) indicated significant difference among these studies. Interestingly, these studies all claimed that significant improvement was found in CHM and chemotherapy as compared with chemotherapy alone.

### 3.6. Herbs Frequently Used in Breast Cancer

30 studies reported herbs and decoctions. Among them,* Radix Astragalus*,* Rhizoma Atractylodis Macrocephalae*, and* Angelica sinensis* are the most frequently used herbs for breast cancer (Table 2).

## 4. Discussion

Recent studies showed a high prevalence of CAM usage among cancer patients, in particular patients with breast cancer [5, 8, 9, 12, 14, 55]. CHM is an especially popular CAM used for cancer patients, while the efficacy of CHM combined with chemotherapy for patients under breast cancer remains unknown and needs to be further explored due to the language barrier because many studies were published in Chinese language. This study conducted a meta-analysis to statistical analysis of the results from individual studies for the purpose of integrating the findings. In the study, the pooled data has shown that CHM combined with chemotherapy significantly improved the tumor response and performance status of breast cancer patients. Also, we found that the combined therapy significantly decreases adverse events caused by chemotherapeutic interventions as compared with chemotherapy alone, including nausea and vomiting at toxicity grade of III-IV, WBC reduction at toxicity grade of III-IV, and platelet reduction at toxicity grade of I–IV or III-IV (Figures 6(a), 6(b), 6(c), and 6(d)). The efficacy of CHM as an adjuvant therapy to chemotherapy for breast cancer is in line with the findings from meta-analysis of CHM combined therapy for advanced non-small-cell lung cancer, colorectal cancer, nasopharyngeal carcinoma, and hepatocellular carcinoma, which suggest that chemotherapy combined with CHM has an advantage in various cancers [20, 56–58].

According to Traditional Chinese Medicine (TCM) theory, sickness is caused by the imbalance of Yin and Yang; restoring the balance of Yin and Yang is the key for curing disease. People can have powerful self-healing ability to remove pathogenic factors and regain health when Yin and Yang of human body are balanced. And self-healing power of human body is attached to importance in the treatment of cancer [59].* Radix Astragalus* (73%),* Rhizoma Atractylodis Macrocephalae* (61%),* Angelica sinensis* (48%),* Codonopsis pilosula* (45%), and* Poria cocos* (39%) were identified as the top five frequently used herbs in the study; all of them have the function of tonifying Qi except* Angelica sinensis* have the function of enriching blood and promoting blood flow, which are in line with the commonest symptoms in postoperative breast cancer patients who are undergoing chemotherapy (such as Qi-deficiency, Qi-blood-deficiency, or blood-stasis). For the most frequently used herb* Radix Astragalus*, some studies have demonstrated that it has the efficacy of antitumor, immunoregulation, and immune restoration by stimulating macrophage and natural killer cell activity while inhibiting T-helper cell type 2 cytokines [60–62]. Besides,* Radix Astragalus* can reduce the toxicity induced by cyclophosphamide that is a common used chemotherapeutic medicine [63]. Although the mechanism of the anticancer effects of most CHMs is not fully understood, the effect of stimulating the immune system and reducing the toxicity induced by chemotherapy might be the two major advantages of CHM as an adjunctive therapy in the treatment of breast cancer.

Tumor response, performance status, toxicity induced by chemotherapy, and immunoregulation were the four major outcome measurements in the study. However, not all included studies simultaneously reported all the four outcomes. For example, Li and Han [52] only reported tumor response and chemotoxicity while Huang et al. [45] reported all four outcomes. Despite that, all available data in these studies were analyzed without any subjective selection. CD3 T cell level, CD4 T cell level, CD8 T cell level, and CD4/CD8 ratio are used as measurements for the evaluation of immunoregulation in breast cancer patients. In this study, we analyzed the mean value of CD3, CD4, CD8, and CD4/CD8 ratio between combined therapy group and chemotherapy group. Although results of these measurements showed that there was a significant improvement in patients treated by chemotherapy combined with CHM, the heterogeneity was also significant when we pooled individual studies which might be caused by different chemotherapy regimens used in different studies [16, 25, 38, 39, 45, 48, 54]. Hence, the efficacy of combined therapy in different chemotherapy regimens compared with chemotherapy alone was further analyzed. Patients treated by CHM combined with CTF (CTX/TAX/5-FU) reported a significant rise of CD3 T cell level as compared with CTF alone (MD = 6.53; 95% CI = 4.80–8.26; *P* < 0.00001; *I* ^2^ = 13%; 2 studies; 105 patients). For CD4 T cell level, CD8 T cell level, and CD4/CD8 ratio, CHM combined with CTF and CHM combined with CEF (CTX/EPI/5-FU) both reported a significant improvement, while being with a contradictory result of heterogeneity test. For example, in the comparison of CD4 T cell level, the value of *I* ^2^ in patients treated by CHM combined with CEF was 0%, while that in patients treated by CHM combined with CTF reported was 68%. Similar situations also happened in CD8 T cell level and CD4/CD8 ratio. The variation of heterogeneity test in these studies may be due to the difference of treatment duration and therapeutic dose except that the different chemotherapy regimens are used in different studies.

The study protocol was registered in PROSPERO (International Prospective Register of Systematic Reviews) with registration number CRD42016033965. Although all reviewers in the study received high-quality training of systematic review and we strictly followed the review procedure stated by the Cochrane Collaboration, there were still several limitations in the study. Firstly, most of the included studies did not clearly describe allocation concealment and blinding, which may contribute to overestimate the effect of treatment group and the emergence of bias. Secondly, publication bias may exist in the study because almost all included studies reported the positive results, while some negative results may be selectively unreported and therefore were not included in the present systematic review. Thirdly, most of the included studies did not provide enough information about methodology such as the way to generate random sequence, intention-to-treat analysis, follow-up, and drop-out rate; the methodological flaws may lead to potential biases. Lastly, different interventions are used in the studies such as different chemotherapy regimens, CHM composition (single herb of compound), oral administration and intravenous injection, treatment duration, or dosage, and all these may lead to heterogeneity among the studies.

## 5. Conclusion

The evidence from this systematic review shows that using CHM as an adjuvant therapy to chemotherapy in comparison with chemotherapy alone has advantages in breast cancer patients. However, particular attention should be paid for applying appropriate and scientific research methodologies to explore CHM as a holistic system because of the complex nature of CHM interventions [64]. Moreover, due to the small sample size, the findings of this meta-analysis may not apply to all patients with breast cancer. Therefore more RCTs with high-quality and large scale are worth performing to investigate the other potential interest of CHM as adjuvant therapy in breast cancer patients, such as survival rate, recurrence rate, and local and distant metastasis, shorten the course of chemotherapy, and so forth.

## Figures and Tables

**Table 1:** Characteristics of the included studies.

Study	Number of participants (TG/CG)/number of drop-outs (TG/CG)	Duration (week)	Treatment group intervention	Control group intervention	CHM formula and ingredients	Assessment of outcomes
Liang et al. 2015 [17]	98 (48 + 50)/0	24	CHM + chemotherapy	Chemotherapy: TAC: TAX/ADM/CTX, or TA: TAX/ADM, or GT: GCB/TAX, or EC: EPI/CTX	Huaier Granule (did not mention specific ingredient)	Survival rate, CD4, CD4/CD8, IL-2, chemotoxicity

Du et al. 2015 [18]	60 (30 + 30)/0	16	CHM + FAC	FAC: 5-FU/ADM/CTX	Modified Xiaoyao powder (*Radix Bupleuri, Angelica sinensis, Radix Paeoniae Alba, Roasted Rhizoma Atractylodis Macrocephalae, Poria cocos, Ginger, Mint, Radix Liquiritiae*)	KPS, chemotoxicity, survival rate, recurrence rate, clinical symptoms

Li 2015 [24]	85 (44 + 41)/0	9	CHM + CEF	CEF: CTX/EPI/5-FU	Fuzheng Xiaoliu compound (*Radix Astragalus, Codonopsis pilosula, Fructus Lycii, Scutellaria barbata, Agrimonia pilosa, Pleione bulbocodioides, Curcuma Zedoary, Rhizoma Sparganii, Coix Seed, Rhizoma, Pericarpium Citri Reticulatae, Licorice Roots Northwest Origin*)	Tumor response, chemotoxicity, serum tumor marker

Li and Han 2015 [52]	60 (30 + 30)/0	12	CHM + TAC	TAC: TAX/ADM/CTX	Yiqihuoxue compound (*Radix Astragalus, Coix Seed, Radix Pseudostellariae, Peach Kernel, Flos Carthami, Rhizoma Atractylodis Macrocephalae, Angelica sinensis, Red Peony Root, Cowherb Seed, Pericarpium Citri Reticulatae, Radix Liquiritiae*)	RECIST, KPS

Chen et al. 2015 [25]	60 (28 + 29)/3	6	CHM + GP	GP: GCB/PDD	Sugan Jianpi Sanjie compound (*Radix Astragalus, Radix Bupleuri, Pericarpium Citri Reticulatae, Radix Curcumae, Pleione bulbocodioides, Rhizoma Atractylodis Macrocephalae, Coix Seed, Oldenlandia diffusa, Scutellaria barbata, Semen Raphani, Polygonatum Kingianum, Radix Liquiritiae*)	Chemotoxicity, KPS, CD3, CD4, CD4/CD8, serum tumor marker

Wang 2015 [26]	60 (30 + 30)/0	4	CHM + FD	FD: 5-FU/DDP	Xiaozheng compound (*Radix Astragalus, Codonopsis pilosula, Oldenlandia diffusa, Scutellaria barbata, Pericarpium Citri Reticulatae Viride, Akebia Fruit, Fructus Aurantii Immaturus, Cortex Magnoliae Officinalis, Tangerine Seed*)	Tumor response, chemotoxicity, survival rate

Fang and Jia 2015 [27]	30 (16 + 14)/0	12	CHM + CE	CE: CTX + EPI	Chinese herbal compound (*Radix Astragalus, Codonopsis pilosula, Roasted Rhizoma atractylodis macrocephalae, Poria cocos, Peach Kernel, Flos Carthami, Red Peony Root, Radix Paeoniae Alba, Radix Isatidis, Nidus Vespae, Dried Radix Rehmanniae, Angelica sinensis, Caulis Spatholobi*)	Tumor response, chemotoxicity, KPS

Bai and Wu 2014 [28]	64 (32 + 32)/0	12	CHM + TEC	TEC: TAX/EPI/CTX	Jinlong capsule (did not mention specific ingredient)	Tumor response, chemotoxicity, KPS

Guo 2014 [29]	76 (38 + 38)/0	8	CHM + CAF	CAF: CTX/ADM/5-FU	Fuzheng Quji decoction (*Radix Astragalus, Caulis Spatholobi, Rhizoma Sparganii, Concha Ostreae, Curcuma Zedoary, Turtle Shell, Sargassum, Roasted Rhizoma Atractylodis Macrocephalae, Polygonatum Kingianum, Codonopsis pilosula, Endothelium Corneum Gigeriae Galli, Amomum villosum, Dried Lacquer, Angelica sinensis, Fructus Lycii*)	Tumor response, chemotoxicity,

Xie 2014 [30]	90 (45 + 45)/0	18	CHM + CAF	CAF: CTX/ADM/5-FU	Shenqifuzheng injection (*Radix Astragalus, Codonopsis pilosula*)	Tumor response, chemotoxicity, peripheral blood lymphocytes

Yang et al. 2014 [31]	86 (43 + 43)/0	2	CHM + TA	TAX/ADM	The beginning of chemotherapy: Xiangsha Liujunzi decoction (*Rhizoma Atractylodis Macrocephalae, Codonopsis pilosula, Rhizoma, Pericarpium Citri Reticulatae, Poria cocos, Chinese Yam, Semen Raphani, Coke Hawthorn Coke Malt, Coke Medicated Leaven, Caulis Bambusae in Taeniam, Purple Perilla, Amomum villosum, Costustoot*); the middle-late stages of chemotherapy: Liuweidihuang decoction (*Poria cocos, Chinese Yam, Rhizoma Atractylodis Macrocephalae, Cornus officinalis, Shorthorned Epimedium, Semen Cuscutae, Mulberry, Radix Rehmanniae Preparata, Rhizoma Alismatis, Caulis Spatholobi, Radix Astragalus, Polygonatum Kingianum*)	Tumor response, chemotoxicity

Cheng et al. 2013 [32]	40 (20 + 20)/0	3	CHM + CAP	CAP: CTX/ADM/PDD	Modified Lizhong decoction (*Red Ginseng, Dried Ginger, Rhizoma Atractylodis Macrocephalae, Radix Liquiritiae, Cornus officinalis, Fructus Schizandrae, Poria cocos, Radix Paeoniae Alba*)	Chemotoxicity, KPS

Wang 2013 [33]	76 (38 + 38)/0	2	CHM + CAF	CAF: CTX/ADM/5-FU	Shenqifuzheng injection (*Radix Astragalus, Codonopsis pilosula*)	Tumor response, chemotoxicity, KPS

Xiong 2012 [34]	48 (24 + 24)/0	6	CHM + CN	CN: CAP + NDP	Fuzheng Xiaoji compound (*Radix Astragalus, Polygonatum Kingianum, Codonopsis pilosula, Rhizoma Atractylodis Macrocephalae, Angelica sinensis, Fructus Lycii, Tortoiseshell, Endothelium Corneum Gigeriae Galli, Caulis Spatholobi, Sargassum, Concha Ostreae*)	RECIST, KPS, serum tumor marker

Zhang and Dang 2012 [35]	60 (30 + 30)/0	4	CHM + NP	NP: NDP/PDD	Fuzheng Guben compound (*Codonopsis pilosula, Curcuma Zedoary, Coix Seed, Coke Hawthorn, Coke Medicated Leaven, Endothelium Corneum Gigeriae Galli, Roasted Rhizoma Atractylodis Macrocephalae, Radix Astragalus, Rhizoma, Pericarpium Citri Reticulatae, Radix Paeoniae Alba, Radix Liquiritiae*)	RECIST, KPS, chemotoxicity

Pan 2011 [36]	160 (80 + 80)/0	4	CHM + CAF	CAF: CTX/ADM/5-FU	Cinobufagin	KPS, chemotoxicity

Liu et al. 2011 [53]	66 (31 + 35)/0	24	CHM + CAF	CAF: CTX/ADM/5-FU	Tianzhicao capsule (did not mention specific ingredient)	KPS, chemotoxicity

Liu et al. 2011 [54]	50 (25 + 25)/0	6	CHM + TE	TE: TAX/EPI	Renshen Yangrong decoction (*Codonopsis pilosula, Radix Astragalus, Radix Rehmanniae Preparata, Radix Paeoniae Alba, Poria cocos, Pericarpium Citri Reticulatae, Rhizoma Atractylodis Macrocephalae, Angelica sinensis Fructus Schizandrae, Polygala tenuifolia, Radix Liquiritiae, Cinnamon, Ginger, Jujube*)	KPS, CD3, CD4, CD4/CD8, CD8, clinical symptoms

Yang 2011 [37]	64 (32 + 32)/0	12	CHM + TA	TA: TAX/ADM	Shenxiao Gualousan (*Radix Bupleuri, Corydalis Tuber, Snakegourd Fruit, Fritillary Bulb, Angelica sinensis, Scrophulariae, Phillyrin, Sargassum, Laminaria, Olibanum, Commiphora myrrha*)	Tumor response, chemotoxicity

Cheng 2011 [38]	50 (26 + 24)/0	8	CHM + TP	TP: TAX/ PDD	Fuzheng Kanai compound (*Radix Astragalus, Angelica sinensis, Radix Pseudostellariae, Roasted Rhizoma Atractylodis Macrocephalae, Poria cocos, Coix Seed, Shorthorned Epimedium, Cornus officinalis, Radix Bupleuri, Rhizoma Cyperi, Oldenlandia diffusa, Curcuma Zedoary, Radix Liquiritiae*)	Tumor response, KPS, CD3, CD4, CD8, clinical symptoms

Zhang et al. 2011 [39]	45 (23 + 22)/0	6	CHM + CTF	CTF: CTX/TAX/5-FU	Xiaoyan decoction (*Codonopsis pilosula, Radix Astragalus, Ligustrum lucidum *Ait.,* Radix Curcuma, Radix Curcumae, Sophora flavescens, Oldenlandia diffusa*)	KPS, CD3, CD4, CD8, CD4/CD8, serum tumor marker, chemotoxicity

Feng et al. 2010 [40]	60 (32 + 28)/0	3	CHM + NFL	NFL: MTX/CF/5-FU	Wu Mei Wan (*Concha Ostreae, Fructus Mume, processed Radix Aconiti Lateralis, Dried Ginger, Cassia Twig, Capsicum annuum, Radix Liquiritiae, Amomum villosum*)	Tumor response, KPS

Zhang 2009 [41]	50 (26 + 24)/0	4	CHM + NP	NP: NDP/PDD	Chinese herbal compound (*Radix Astragalus, Oldenlandia diffusa, Nidus Vespae, Angelica sinensis, Radix Pseudostellariae, Roasted Rhizoma Atractylodis Macrocephalae, Poria cocos, Coix Seed, Cornus officinalis, Achyranthes bidentata, Radix Bupleuri, Radix Paeoniae Alba, Curcuma, Pericarpium Citri Reticulatae, Rhizoma, Fructus Aurantii*)	Tumor response, clinical symptoms, KPS, chemotoxicity

Yang et al. 2008 [42]	59 (28 + 27)/4	12	CHM + NP	NP: NDP/PDD	Guben Yiliu II (*Codonopsis pilosula, Poria cocos, Rhizoma Atractylodis Macrocephalae, Radix Astragalus, Ligustrum lucidum *Ait.,* Fructus Lycii, Shorthorned Epimedium, Rhizoma Chuanxiong, Caulis Spatholobi, Curcuma Zedoary, Fritillary Bulb, Sophora flavescens*)	Tumor response, KPS, serum tumor marker, clinical symptoms, chemotoxicity

Hu et al. 2008 [43]	52 (28 + 24)/0	20 d	CHM + CAF	CAF: CTX/ADM/5-FU	Yiqihuoxue decoction (*Codonopsis pilosula, Radix Astragalus, Angelica sinensis, Colla Corii Asini, Pseudoginseng, Peach Kernel, Flos Carthami, Rhizoma Atractylodis Macrocephalae, Coix Seed, Fructus Lycii, Eucommia ulmoides, Fritillary Bulb, Selfheal*)	Tumor response, chemotoxicity

Chen et al. 2008 [16]	60 (30 + 30)/0	24	CHM + CEF	CEF: CTX/EPI/5-FU	Dangguibuxue decoction (*Radix Astragalus, Angelica sinensis*)	Quality of life, chemotoxicity, CD3, CD4, CD8, CD4/CD8

Tang et al. 2007 [44]	60 (30 + 30)/0	12	CHM + CEF	CEF: CTX/EPI/5-FU	Yiqijiedu decoction (*Radix Astragalus, Angelica sinensis, Caulis Spatholobi, Ligustrum lucidum *Ait.,* Selfheal, Radix Bupleuri, Coke Hawthorn, Coke Malt, Coke Medicated Leaven*)	Clinical symptoms, CD4, CD8, CD4/CD8, KPS

Huang et al. 2007 [45]	60 (30 + 30)/0	6	CHM + CTF	CTF: CTX/TAX/5-FU	Jianpi Xiaoji decoction (*Radix Pseudostellariae, Radix Astragalus, Coix Seed, Rhizoma Atractylodis Macrocephalae, Poria cocos, Oldenlandia diffusa, Fructus Aurantii, Pericarpium Citri Reticulatae, Radix Liquiritiae*)	Tumor response, KPS, chemotoxicity, CD3, CD4, CD8, CD4/CD8

Yang et al. 2007 [46]	38 (20 + 18)/0	9	CHM + CEF	CEF: CTX/EPI/5-FU	Taohongsiwu decoction (*Radix Paeoniae Alba, Rhizoma Chuanxiong, Angelica sinensis, Flos Carthami, Radix Rehmanniae Preparata, Peach Kernel*)	Tumor response, clinical symptoms, chemotoxicity

Dai et al. 2004 [47]	46 (25 + 21)/0	6	CHM + NP	NP: NDP/PDD	Tiaoganyangxue compound (*Radix Bupleuri, Dried Radix Rehmanniae, Rhizoma Cyperi, Pericarpium Citri Reticulatae Viride, Pericarpium Citri Reticulatae, Rhizoma Chuanxiong, Angelica sinensis, Radix Paeoniae Alba, Poria cocos, Rhizoma Atractylodis Macrocephalae, Radix Astragalus, Radix Pseudostellariae, Snakegourd Fruit, Fritillary Bulb, Concha Ostreae, Uniflower Swisscentaury Root, Cowherb Seed, Stir-baked Squama Manitis, Dandelion*)	Tumor response, chemotoxicity, KPS

Li et al. 2004 [48]	75 (40 + 35)/0	12	CHM + NE	NE: NDP/ EPI	Shenqifuzheng injection (*Codonopsis pilosula, Radix Astragalus*)	Tumor response, chemotoxicity

Yang and Sun 2004 [49]	51 (27 + 24)/0	9	CHM + CMF	CMF: CTX/MTX/5-FU	Modified Liujunzi decoction (*Codonopsis pilosula, Poria cocos, Rhizoma Atractylodis Macrocephalae, Pericarpium Citri Reticulatae, Rhizoma, Radix Liquiritiae, Radix Astragalus, Angelica sinensis, Radix Rehmanniae Preparata, Ligustrum lucidum *Ait.,* Morinda officinalis, Fructus Psoraleae*)	Cellular immune function, tumor response, chemotoxicity

Huang et al. 2003 [50]	66 (37 + 29)/0	6	CHM + CMF	CMF: CTX/MTX/5-FU	Modified Bazhen decoction (*Radix Rehmanniae Preparata, Angelica sinensis, Rhizoma Atractylodis Macrocephalae, Poria cocos, Rhizoma Chuanxiong, Radix Paeoniae Alba, Codonopsis pilosula, Radix Astragalus, Radix Liquiritiae, Ligustrum lucidum *Ait.,* Pericarpium Citri Reticulatae*)	Tumor response, KPS, chemotoxicity, clinical symptoms

TG = treatment group; CG = control group; CHM = Chinese herbal medicine; KPS = Karnofsky performance scale; RECIST = Response Evaluation Criteria In Solid Tumors; TAC = paclitaxel + adriamycin + cyclophosphamide; TA = paclitaxel + adriamycin; GT = gemcitabine + paclitaxel; EC = epirubicin + cyclophosphamide; NE = nedaplatin + epirubicin; FAC = fluorouracil + adriamycin + cyclophosphamide; CEF = cyclophosphamide + epirubicin + fluorouracil; GP = gemcitabine + cisplatinum; FD = fluorouracil + cisplatin; CE = cyclophosphamide + epirubicin; TEC = paclitaxel + epirubicin + cyclophosphamide; CAF = cyclophosphamide + adriamycin + fluorouracil; CAP = cyclophosphamide + adriamycin + *cis*-platinum; CN = cyclophosphamide + nedaplatin; NP = nedaplatin + *cis*-platinum; TE = paclitaxel + epirubicin; TP = paclitaxel + *cis*-platinum; CTF = cyclophosphamide + paclitaxel + fluorouracil; NFL = methotrexate + cyclophosphamide + fluorouracil; CMF = cyclophosphamide + methotrexate + fluorouracil.

**Table 2:** Herbs frequently used for breast cancer.

Chinese herbal medicine	Frequency	
	Count	%
*Radix Astragalus*	24	73%
*Rhizoma Atractylodis Macrocephalae*	20	61%
*Angelica sinensis*	16	48%
*Codonopsis pilosula*	15	45%
*Poria cocos*	13	39%
*Radix Liquiritiae*	12	36%
*Pericarpium Citri Reticulatae*	11	33%
*Radix Paeoniae Alba*	9	27%
*Coix Seed*	8	24%
*Radix Bupleuri*	7	21%
